# A robot scheduling method based on rMAPPO for H-beam riveting and welding work cell

**DOI:** 10.1371/journal.pone.0331515

**Published:** 2025-09-04

**Authors:** Jianbin Zheng, Chuyi Zhou, Yang Gao, Ziyao Chen, Yifan Gao, Yizhuo Zhang, Xinyu Zhou, Yuanzheng Ou

**Affiliations:** Hubei Key Laboratory of Broadband Wireless Communication and Sensor Networks, School of Information Engineering, Wuhan University of Technology, Wuhan, Hubei, China; Industrial University of Ho Chi Minh City, VIET NAM

## Abstract

The H-beam riveting and welding work cell is an automated unit used for processing H-beams. By coordinating the gripping and welding robots, the work cell achieves processes such as riveting and welding stiffener plates, transforming the H-beam into a stiffened H-beam. In the context of intelligent manufacturing, there is still significant potential for improving the productivity of riveting and welding tasks in existing H-beam riveting and welding work cells. In response to the multi-agent system of the H-beam riveting and welding work cell, a recurrent multi-agent proximal policy optimization algorithm (rMAPPO) is proposed to address the multi-agent scheduling problem in the H-beam processing. The algorithm employs recurrent neural networks to capture and process historical information. Action masking is used to filter out invalid states and actions, while a shared reward mechanism is adopted to balance cooperation efficiency among agents. Additionally, value function normalization and adaptive learning rate strategies are applied to accelerate convergence. This paper first analyzes the H-beam processing flow and appropriately simplifies it, develops a reinforcement learning environment for multi-agent scheduling, and applies the rMAPPO algorithm to make scheduling decisions. The effectiveness of the proposed method is then verified on both the physical work cell for riveting and welding and its digital twin platform, and it is compared with other baseline multi-agent reinforcement learning methods (MAPPO, MADDPG, and MASAC). Experimental results show that, compared with other baseline methods, the rMAPPO-based agent scheduling method can reduce robot waiting times more effectively, demonstrate greater adaptability in handling different riveting and welding tasks, and significantly enhance the manufacturing efficiency of stiffened H-beam.

## 1 Introduction

The H-beam has become one of the most widely used steel components in building and industrial structures due to its excellent bending resistance [1,2]. The stiffener plate is a reinforced structural component that enhances the local stiffness of the H-beam. Typically, the stiffener plate is welded onto the web or flange of the H-beam to improve structural strength [3], prevent local buckling, and enhance load distribution efficiency. An H-beam with welded stiffener plates is referred to as a stiffened H-beam. As a widely used structural component in construction, the stiffened H-beam plays a crucial role in load bearing and improving structural stability.

The traditional stiffened H-beam production process mainly relies on semi-automated equipment or manual operations, leading to low production efficiency and difficulties in coordinating the riveting and welding processes, making it challenging to meet high-efficiency production demands. As intelligent manufacturing drives the transformation of traditional manufacturing towards high-end and intelligent production [4], an automated and integrated work cell, combining riveting and welding with a multi-robot system collaborating on these tasks, is highly valuable [5–7]. The H-beam riveting and welding work cell is mainly composed of a gripping robot and multiple welding robots, all mounted on their respective linear tracks. A scheduling method plans the movement of each robot base along the track. Once the robots reach their designated positions, the gripping and welding robots execute precise operations, such as gripping, riveting, and welding stiffener plates, using teaching programming [8], thereby completing the production process of the stiffened H-beam.

In a multi-robot system, due to environmental uncertainties and task allocation, complex multi-robot collaboration problems can be formulated as multi-agent scheduling problems [9,10]. The objective of multi-agent scheduling is to improve the production efficiency of the system by effectively allocating and optimizing robotic resources while meeting task requirements. To address multi-agent scheduling problems, many researchers have proposed a range of solutions using traditional optimization scheduling algorithms. B. Zhou et al. proposed a scheduling framework for multi-station multi-welding robots, which employs a hierarchical optimization algorithm to achieve efficient robot scheduling and task allocation, ultimately improving welding efficiency in real-world production [11]. A. Casalino et al. introduced a scheduling method using time Petri nets, which optimizes assembly task planning for collaborative automated systems by leveraging runtime data and minimizes idle time in human-robot collaboration [12]. Y. Wang et al. applied a multi-objective evolutionary algorithm incorporating tabu search, a non-dominated sorting rule, and individual population density into a scheduling optimization model for a multi-station robotic welding system, enhancing batch production efficiency in factories [13]. Although traditional optimization scheduling algorithms perform well in small-scale, well-structured production environments, they remain limited in handling dynamic changes, and multi-agent collaboration due to their lack of adaptability, scalability, and intelligence.

The existing H-beam riveting and welding work cell, as a multi-agent system, still has significant potential for reducing multi-agent waiting times and improving the productivity of riveting and welding tasks. Given the limitations of traditional scheduling methods, this paper constructs a two-dimensional interactive environment for multi-agent scheduling. A recurrent multi-agent proximal policy optimization (rMAPPO) algorithm is designed accordingly. The rMAPPO algorithm is developed for fully cooperative scenarios and adopts a centralized training and decentralized execution (CTDE) framework. Recurrent neural networks are used to process historical information and guide optimal scheduling decisions. A shared reward mechanism is introduced to improve overall coordination efficiency. Action masking is applied to eliminate invalid states and actions, and both value function normalization and adaptive learning rate optimization are used to accelerate convergence. Furthermore, a H-beam riveting and welding work cell and its digital twin platform are built to experimentally validate the effectiveness of the proposed algorithm. The main contributions of this paper are as follows:

1. Designed a rMAPPO-based multi-agent reinforcement learning scheduling algorithm for fully cooperative scenarios. A multi-agent interactive environment is established, with a customized reward function designed to guide learning. By incorporating shared rewards, value normalization, action masking, and adaptive learning rate optimization, the coordination efficiency of robots in the H-beam riveting and welding workstation is improved.
2. Developed a physical H-beam riveting and welding work cell and its digital twin platform, utilizing digital twin technology to establish a connection between the physical and cyber space, thereby enabling intelligent monitoring of the physical work cell.
3. Validated the scheduling decisions derived from the multi-agent interaction environment through experiments conducted on both the physical work cell and the digital twin platform. Additionally, comparative experiments were performed to demonstrate the effectiveness of the proposed method in both efficiency and stability.

The remainder of this paper is structured as follows: section 2 reviews the related work; section 3 introduces the system framework; section 4 provides a detailed explanation of the theoretical foundations of rMAPPO; section 5 details the experimental design and results; and section 6 is the conclusion.

## 2 Related work

This section reviews reinforcement learning-based multi-agent scheduling methods and provides an overview of the application of digital twins in combination with reinforcement learning in intelligent manufacturing.

Multi-agent reinforcement learning scheduling methods enable agents to interact with the environment, make decisions at each timestep, and continuously learn and adapt, ultimately forming an optimal policy with the goal of achieving the system’s predefined objectives [14,15]. Trained via reinforcement learning, the scheduling system supports autonomous decision-making, with self-adaptive and self-learning capabilities, thereby improving overall system intelligence. It is applicable to task allocation, scheduling, and path planning tasks [16]. In recent years, numerous studies have explored reinforcement learning-based scheduling problems. C.-L. Liu et al. proposed a deep reinforcement learning method based on the Actor-Critic (AC) framework for the dynamic job shop scheduling problem, incorporating asynchronous updates and deep deterministic policy gradient (DDPG) to train the model, making it suitable for dynamic production environments [17]. T. Zhou et al. proposed an online scheduling method for smart factories based on multi-agent reinforcement learning (MARL). By employing a distributed AI scheduling architecture and federated learning mechanisms, and integrating Internet of Things (IoT)-based data transmission, their method not only improved the production efficiency of smart factories but also optimized the workload balance among agents [18]. B. Kruekaew et al. combined Q-learning with the artificial bee colony algorithm and proposed a reinforcement learning-based hybrid artificial bee colony task scheduling algorithm. Experimental results demonstrated its strong adaptability in task scheduling optimization and load balancing in cloud computing environments [19]. Y. Zhang et al. proposed a multi-agent scheduling method for manufacturing systems based on deep reinforcement learning. This method integrates the proximal policy optimization (PPO) algorithm, the contract net protocol task allocation mechanism, and edge computing to address dynamic job shop scheduling problems. Experimental results demonstrate that it outperforms traditional approaches in scheduling efficiency, convergence speed, and robustness [20]. J.-D. Zhang et al. introduced a solution for flexible job shop scheduling called DeepMAG. DeepMAG integrates deep reinforcement learning into MARL and constructs a graph structure to represent the relationships between tasks and machines, allowing agents to collaborate more effectively and enhance scheduling performance [21]. M. Wang et al. proposed a resilient scheduling framework for scheduling in multi-robot, multi-station welding pipeline workshops. The framework consists of proactive scheduling and a deep reinforcement learning-based recovery scheduling method. Experimental results demonstrate that this approach effectively optimizes welding task allocation and enhances the robustness of scheduling strategies [22]. H. Kang et al. applied the multi-agent proximal policy optimization (MAPPO) algorithm to solve the UAV resource scheduling problem in hierarchical aerial computing systems, enhancing computational efficiency and minimizing computational latency [23]. H. Zeng et al. proposed a collaborative UAV scheduling framework based on the Actor-Critic architecture. By periodically updating the decision network and real-time scheduling policy through cooperative mechanisms, the framework achieves superior performance in dynamic and complex environments [24]. X. Wang et al. proposed a solution for cloud manufacturing scheduling in dynamic environments by developing a multi-agent graph convolution integrated scheduler that incorporates the graph convolution network. Multiple experimental scenarios demonstrated the effectiveness and generalization capability of the proposed method [25].

Digital twin technology enables high-fidelity simulation and real-time monitoring by constructing virtual models of physical systems and is widely used in intelligent manufacturing [26,27]. The integration of reinforcement learning and digital twins provides an efficient, safe, and intelligent approach to optimization in intelligent manufacturing. J. Liu et al. addressed the challenge of dynamic planning for robotic disassembly sequences under incomplete information by establishing a digital twin model of the disassembly process. They combined it with deep Q-network (DQN) to derive solutions and validated its effectiveness using a digital twin platform [28]. K. Xia et al. proposed a digital twin-enhanced task scheduling method that incorporates DQN. The digital twin serves as a high-fidelity simulation environment, allowing the reinforcement learning agent to learn the optimal scheduling strategy in the virtual space before deployment in the physical system [29]. G. Shen et al. proposed a digital twin-based deep reinforcement learning framework incorporating the behavior-coupled deep deterministic policy gradient (BCDDPG) algorithm for optimizing the motion of multi-UAV swarms. Experimental results demonstrated the efficiency and stability of this method in UAV task execution, and it is also applicable to swarm robotic scheduling [30]. In summary, the integration of reinforcement learning and digital twin technology enhances system intelligence. The simulation environment constructed using digital twin technology provides an efficient, safe, and controllable platform for experimental validation and testing in reinforcement learning.

## 3 System framework

This section first introduces the production workflow of the H-beam riveting and welding work cell along with its corresponding digital twin representation. Then, the characteristics of the actual production process are analyzed, appropriately simplified, and the underlying multi-agent scheduling problem is identified. A reinforcement learning environment for multi-agent interaction is developed to address this challenge. Finally, the overall system framework integrating the rMAPPO algorithm and the digital twin is presented.

### 3.1 Physical riveting and welding work cell and digital twin platform

**Fig 1:**
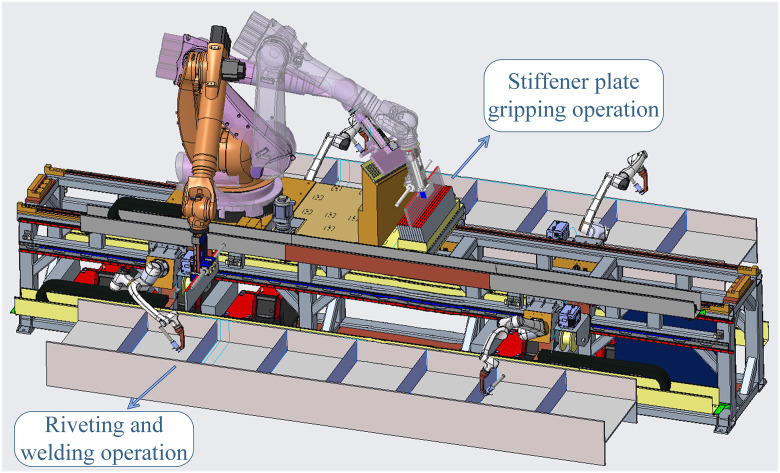
Production workflow of stiffened H-beams.

During the production of stiffened H-beams in the H-beam riveting and welding work cell, stiffener plate gripping and riveting, along with the subsequent welding operation, are critical steps in the production workflow. As shown in Fig 1, the gripping robot first executes the stiffener plate gripping operation and then transports the plate to one side of the H-beam for riveting. After the stiffener plate is riveted to the H-beam, the welding robot performs the welding operation at the designated position to secure it. To complete the riveting and welding of a single stiffener plate, the gripping robot collaborates with a welding robot for a specific duration. Upon completing the collaboration, the gripping robot moves to the next designated position, carrying another stiffener plate, and collaborates with a nearby welding robot to perform the riveting and welding operations. This process continues until the entire production sequence is fully executed.

**Fig 2:**
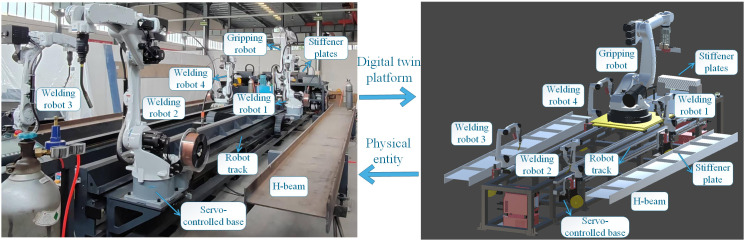
Illustration of the physical riveting and welding work cell and digital twin platform.

**Fig 3:**
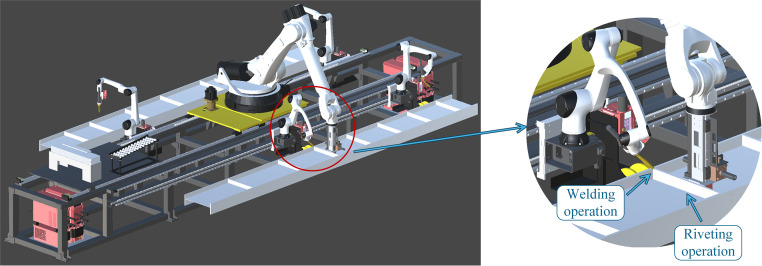
Demonstration of stiffener plate riveting and welding operations.

The physical H-beam riveting and welding work cell and its digital twin platform are illustrated in Fig 2. The system primarily consists of a gripping robot that grips and rivets the stiffener plates, as well as four welding robots. The gripping robot, along with the welding robots on both sides, is equipped with servo-controlled bases, allowing them to traverse along the robot track. On both sides of the work cell, H-beams are securely positioned. Stiffener plates are pre-stacked at the front of the work cell. This enables the gripping robot to pick up a new plate from the stacking area after completing the previous riveting and welding task. In the H-beam riveting and welding work cell, the gripping, riveting, and welding of stiffener plates are performed by the respective robots at designated positions according to pre-recorded teaching instructions. The stiffener plate riveting and welding operations simulated on the digital twin platform are illustrated in Fig 3. Once the gripping robot and a welding robot complete a riveting and welding cycle, the gripping robot returns to its initial position. It then picks up a new stiffener plate and moves to the next designated position to collaborate on the subsequent task. This process continues until both H-beams are completely assembled. Once the process is completed, all robots return to their initial positions, marking the end of the production sequence.

Within the digital twin platform of the work cell, the steel structure framework (excluding the robots) was modeled in SolidWorks. The positions of all objects in the model were determined based on precise measurements. According to the measurement data, their spatial positions in cyber space were determined using global or local coordinates. Additionally, the 3D models of the robots were defined in unified robot description format (URDF) files. Collision detection and feedback are enabled by integrating collision boxes into the robot model. Finally, the entire digital twin platform was developed in Unity 3D and connected to the physical work cell via an industrial computer.

**Fig 4:**
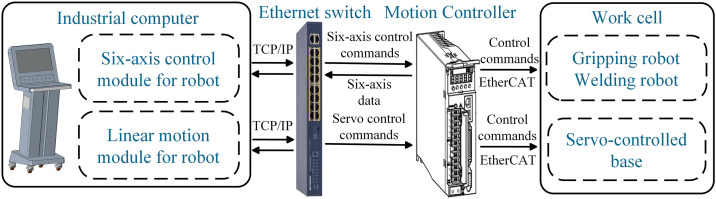
The connection of physical space.

For data transmission from the physical work cell to the digital twin platform, six-axis data is acquired via socket communication [31]. This data is then used to control the corresponding digital twin robots in real time through C# scripts. The industrial computer controls the physical work cell and facilitates data exchange between the two systems. It serves as the master station, while the gripping and welding robots function as slave stations. The master station transmits request frames to slave stations over IP, which, upon reception, execute the corresponding operations. As shown in Fig 4, the industrial computer communicates with a 16-port industrial Ethernet switch via the TCP/IP protocol. It transmits six-axis control commands and receives real-time six-axis data through this connection. After configuration, the Ethernet switch establishes a data link layer connection with the motion controller. Motion commands are transmitted from the motion controller to the robot layer via the EtherCAT protocol, enabling precise control of the servo-controlled base and robot posture.

**Fig 5:**
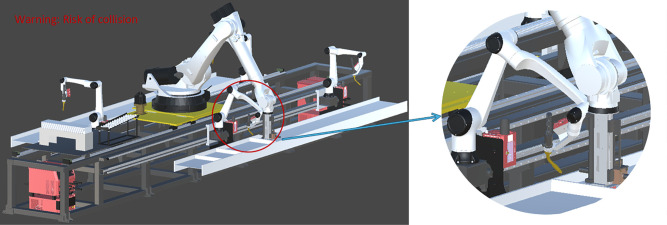
Collision risk warning.

By leveraging digital twin technology to link the physical and virtual spaces, intelligent monitoring of the production process at the physical work cell is enabled. Upon detecting equipment failures or other anomalies, the digital twin platform identifies collision risks, as illustrated in Fig 5. It immediately issues an alert, prompting the industrial computer to control the physical work cell to stop production and await manual intervention. Production resumes only after the fault is cleared.

### 3.2 Development of multi-agent interactive environment and system framework

During actual production, while maintaining safe and stable operations, the operating speed of each servo-controlled base is set to the highest feasible fixed value $v_{max}$, enabling robots to reach their target positions in the shortest possible time. Let $T_{p}$ denote the total production time. Since the riveting and welding time $T_{w}$ starts after the gripping and welding robots reach their designated positions, it is influenced by the process itself. Optimizing $T_{w}$ without compromising quality is challenging. Therefore, to optimize production efficiency, adjusting the scheduling time $T_{s}$ of each robot is the most effective approach.


$$\text{m}\text{in}\left(T_{p}\right)=\text{m}\text{in}\left(T_{w}+T_{s}\right)=T_{w}+\text{m}\text{in}\left(T_{s}\right).$$ (1)

**Fig 6:**
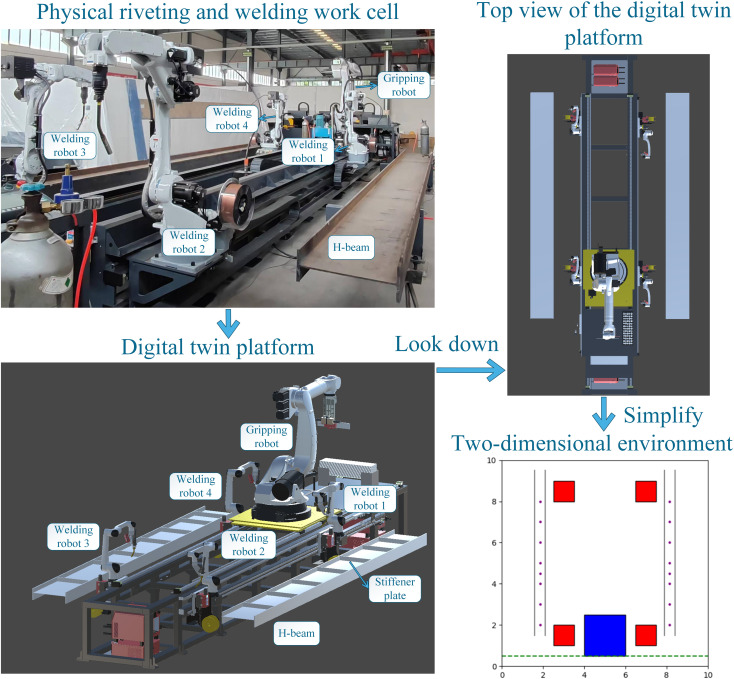
Abstraction of the 3D scene into a 2D multi-agent interactive environment.

During riveting and welding operations, the servo-controlled bases of both the gripping and welding robots remain stationary, while their six-axis arms perform active movements. During the gripping operation, the gripping robot grips the stiffener plate at its initial position, with active six-axis arm movements. During the scheduling process, the servo motors of each robot base move, while the six-axis joints of all robots remain in their respective zero-position configurations. The key to optimizing multi-robot collaborative scheduling lies in optimizing the movement of the servo-controlled bases of the five robots. Therefore, in constructing the multi-agent reinforcement learning environment, as shown in Fig 6, only a 2D top view is considered. The 3D model of the work cell is transformed into a 2D top view multi-agent interactive environment.

Fig 6 illustrates the 2D environment for multi-agent reinforcement learning. The blue square represents the gripping robot, while the four red squares represent the welding robots. Each robot is restricted to movement along the y-axis and has a predefined range of motion, which is consistent with the settings in the 3D scene. Collision detection is applied to welding robots sharing the same track. Meanwhile, the two H-beams are positioned on both sides, and purple dots indicate the designated riveting and welding positions. A rule is defined that when the x-coordinates of the gripping robot and a welding robot simultaneously align with that of a welding point, both robots halt at the position. They remain stationary for a fixed number of timesteps to accommodate the execution of the riveting and welding operations. Afterward, the gripping robot returns to its initial position and remains there for a certain number of timesteps to account for the stiffener plate gripping operation during actual operation. Once it has remained at the origin for a certain number of timesteps, the gripping robot resumes its movement and collaborates with another welding robot to perform the next riveting and welding task for a new stiffener plate. Finally, once all stiffener plates have been riveted and welded, all five robots return to their initial positions, marking the end of the production process.

**Fig 7:**
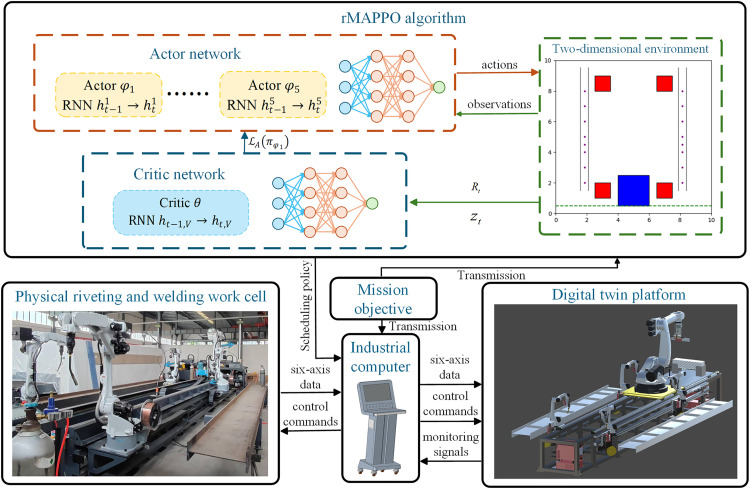
Framework of riveting and welding work cell based on rMAPPO and digital twin.

After appropriate simplification of the problem and construction of the 2D environment, the rMAPPO algorithm is applied to solve the multi-agent scheduling problem. The task is addressed in a continuous action space under a fully cooperative framework. Additionally, it integrates a digital twin system to validate the model’s performance. The overall system framework is illustrated in Fig 7. The rMAPPO network is designed based on the Actor-Critic framework, where the actor networks take the agents’ observations as input and outputs the corresponding actions that the agents need to execute at the current timestep Δ*t*. Each action represents the distance an agent needs to move, ranging from 0 to *v*_*max*_ ∙ Δ*t*. Meanwhile, the critic network evaluates the agents’ actions based on accumulated rewards and global state information, guiding the actor networks to optimize their policies. As shown in Fig 7, after defining the production mission objective for the riveting and welding work cell, the corresponding information is transmitted to the industrial computer and the 2D environment. The trained rMAPPO network generates actions for each agent, which are integrated into a unified scheduling policy for the riveting and welding work cell. This policy is transmitted to the industrial computer and translated into corresponding servo base control commands. The commands are then dispatched to both the digital twin platform and the physical work cell for synchronized execution. The servo-controlled bases of the robots in the physical work cell execute precise movements to reach the designated positions. Upon reaching the target positions, the industrial computer controls the robots to perform the corresponding six-axis motions (such as riveting, gripping, or welding), while concurrently transmitting the six-axis data of the respective robots to the digital twin platform. This enables the corresponding robots in the digital twin platform to replicate the six-axis motions in real time. Ultimately, a cyber-physical co-control mechanism is established between the digital twin platform and the physical work cell. This framework not only enhances production efficiency using the rMAPPO model but also facilitates real-time monitoring of the work cell’s production status. Monitoring signals can be transmitted to the industrial computer by the digital twin platform. The framework also supports offline validation. By using six-axis data from the robot’s teaching motions, the correctness of rMAPPO decisions can be verified within the digital twin platform of the work cell. Once validated, the system can be connected to the physical work cell to achieve a real-time control loop.

## 4 Scheduling method

This section first introduces the proximal policy optimization (PPO) algorithm. Then, it describes the principles and framework of the multi-agent proximal policy optimization (MAPPO) algorithm in the context of optimization. Finally, it presents the network structure and reward function of the recurrent multi-agent proximal policy optimization (rMAPPO) algorithm, which is used to tackle multi-robot scheduling in the riveting and welding work cell.

### 4.1 Proximal policy optimization

The primary objective of reinforcement learning algorithms is to maximize the expectation of the cumulative rewards $J\left(\pi_{\phi }\right)$, which is given by:


$$J\left(\pi_{\phi }\right)=\underset{\phi }{\text{max}}\left\{{𝔼}_{\tau \sim \pi_{\phi }}\left[\sum {\mathrm{\backslash nolimits}}_{t=\text{0}}^{T}\gamma^{t}r(s_{t}\text{,}a_{t})\right]\right\}.$$ (2)

Here, $s_{t}$ and $a_{t}$ represent the agent’s state and action at timestep t, respectively. T represents the maximum number of timesteps for agent exploration. $r(s_{t}\text{,}a_{t})$ is the immediate reward at timestep t, while γ is the discount factor, which controls the trade-off between long-term and short-term rewards. The sequence $\tau =(s_{\text{0}}\text{,}a_{\text{0}}\text{,}s_{\text{1}}\text{,}a_{\text{1}}\ldots )$ defines the agent’s trajectory, consisting of states and actions. $\pi_{\phi }$ represents the agent’s policy with parameters ϕ.

However, the PPO algorithm does not directly maximize the expected return $J\left(\pi_{\phi }\right)$, Instead, it follows the Actor-Critic framework, where the actor uses a neural network to determine the current policy and select the action $a_{t}$, while the critic models the value function $V_{\theta }(s_{t})$ using another neural network, where θ represents the parameters of the value function. The critic assesses whether the action chosen by the actor is optimal or advantageous.

In traditional policy gradient (PG) methods [32], the gradient of the policy $\pi_{\phi }$ is defined as:


$$\nabla_{\phi }J\left(\pi_{\phi }\right)={𝔼}_{t}\left[\nabla_{\phi }log\;\pi_{\phi }(a_{t}|s_{t})\cdot A_{\pi_{\phi }}(s_{t},a_{t})\right].$$ (3)

Here, $A_{\pi_{\phi }}(s_{t}\text{,}a_{t})$ represents the advantage function, which measures the relative advantage of the current action $a_{t}$. In PPO, the actor’s objective function is derived from it and further refined into the objective function ${\text{L}}_{A}\left(\pi_{\phi }\right)$, which is given by:


$${ℒ}_{A}\left(\pi_{\phi }\right)={𝔼}_{t}\left[\text{min}(\ell_{t}(\phi ){\cdot A}_{\pi_{\phi }}(s_{t},a_{t}),\text{clip}(\ell_{t}(\phi ),1-\in ,1+\in ){\cdot A}_{\pi_{\phi }}(s_{t},a_{t}))\right].$$ (4)

PPO iteratively approximates the optimal policy by optimizing the above objective function, and thus indirectly optimizing $J\left(\pi_{\phi }\right)$. It replaces the conventional log probability formulation $\text{log}\;\pi_{\phi }(a_{t}|s_{t})$ with the probability ratio of the new policy to the old policy:


$$\ell_{t}(\phi )=\frac{\pi_{\phi }(a_{t}|s_{t})}{\pi_{\phi }^{\text{old}}(a_{t}|s_{t})}.$$ (5)

This replacement mitigates update instability. The clipping mechanism is controlled by the hyperparameter ∈ to limit the magnitude of policy updates. With a properly chosen clipping coefficient ∈, the return $J\left(\pi_{\phi }\right)$ is non-decreasing [33]. The advantage function $A_{\pi_{\phi }}(s_{t}\text{,}a_{t})$ is computed using the generalized advantage estimation (GAE) method [34], formulated as:


$$A_{\pi_{\phi }}\left(s_{t},a_{t}\right)=\sum {\mathrm{\backslash nolimits}}_{k=0}^{T-t}{\left(\gamma \lambda \right)}^{k}\delta_{t+k}.$$ (6)

Here, λ is a hyperparameter, $\delta_{t}=r\left(s_{t}\text{,}a_{t}\right)+\gamma V_{\theta }\left(s_{t+\text{1}}\right)-V_{\theta }\left(s_{t}\right)$.

In PPO, the critic’s objective function ${ℒ}_{C}(\theta )$ is defined as:


$${ℒ}_{C}(\theta )={𝔼}_{t}\left[{\left(V_{\theta }\left(s_{t}\right)-R_{t}\right)}^{2}\right].$$ (7)

$V_{\theta }\left(s_{t}\right)$ denotes the Critic network’s state value function, and $R_{t}$ represents the cumulative discounted rewards. The critic enhances the precision of state value estimation through minimizing ${ℒ}_{C}(\theta )$.

The complete objective function of PPO, $ℒ\left(\pi_{\phi },\theta \right)$, integrates the objective functions of the actor and critic, while incorporating the policy entropy function $H(\pi_{\phi })$ to promote exploration. The final formulation is:


$$ℒ\left(\pi_{\phi },\theta \right)={ℒ}_{A}\left(\pi_{\phi }\right)-c_{1}{ℒ}_{C}(\theta )+c_{2}H\left(\pi_{\phi }\right),$$ (8)

where $c_{\text{1}}$ and $c_{\text{2}}$ are scaling factors that control the trade-off among different components. In PPO, the policy network (actor) and value network (critic) collaborate to maximize $ℒ\left(\pi_{\phi },\theta \right)$. The policy network updates the policy through maximizing ${ℒ}_{A}(\pi_{\phi })$, while the value network refines state value estimation through minimizing ${ℒ}_{C}(\theta )$.

### 4.2 Multi-agent proximal policy optimization

**Fig 8:**
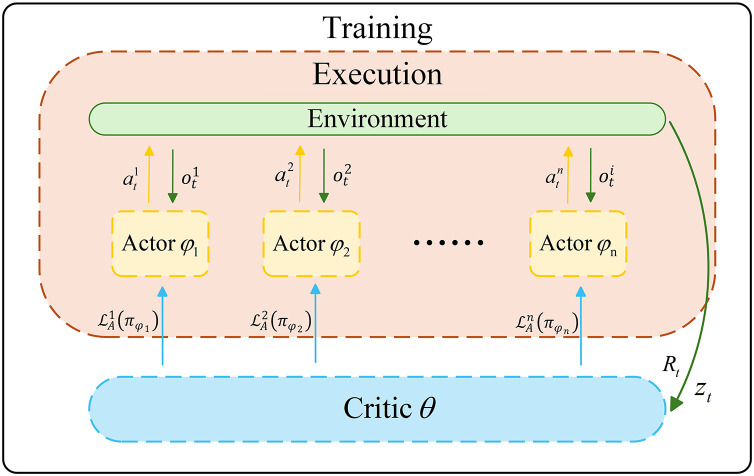
Framework of MAPPO.

MAPPO is an adaptive variant of the PPO algorithm that adapts it for the multi-agent reinforcement learning (MARL) domain [35]. In multi-agent settings, the decision of one agent can affect both the environment observed by other agents and their subsequent decisions [36]. As shown in Fig 8, MAPPO adopts the centralized training and decentralized execution (CTDE) framework. During the training phase, all agents share a centralized critic, which leverages global information $z_{t}$ (including each agent’s observation $o_{t}^{i}$, the action $a_{t}^{i}$ generated by the actor networks) and the discounted reward $R_{t}$. The critic estimates the overall state value based on global information and optimizes the actor’s policy updates using the objective function ${ℒ}_{A}^{i}\left(\pi_{\phi_{i}}\right)$. During the execution phase, each agent independently selects action based solely on its local observation $o_{t}^{i}$, ensuring agent autonomy and scalability.

The core objective of MAPPO is to maximize the expectation of the joint cumulative reward of all agents, $J(\pi_{\varphi })$, formulated as:


$$J\left(\pi_{\varphi }\right)=\underset{\varphi }{\text{max}}\left\{{𝔼}_{\tau \sim \pi_{\varphi }}\left[\sum {\mathrm{\backslash nolimits}}_{i=1}^{n}\sum {\mathrm{\backslash nolimits}}_{t=0}^{T}\gamma^{t}r^{i}(s_{t}^{i},a_{t}^{i})\right]\right\}.$$ (9)

Here, $\pi_{\varphi }$ denotes the joint policy of all agents, and $\varphi =\left\{\phi_{1},\phi_{2},\ldots ,\phi_{n}\right\}$ is the set of parameters defining the joint policy. $s_{t}^{i}$ and $a_{t}^{i}$ denote the state and corresponding action of the *i*-th agent at timestep t, respectively. $r^{i}(s_{t}^{i}\text{,}a_{t}^{i})$ represents the reward received by the *i*-th agent at timestep t.

MAPPO also indirectly optimizes $J(\pi_{\varphi })$ through policy optimization for individual agents. It incorporates a clipping mechanism to constrain the magnitude of policy updates, mitigating instability during training. The objective function for each actor, ${ℒ}_{A}^{i}\left(\pi_{\phi_{i}}\right)$, is defined as:


$${ℒ}_{A}^{i}\left(\pi_{\phi_{i}}\right)=\left[\text{min}\left(\ell_{t}^{i}(\phi_{i}){\cdot A}_{\pi_{\phi_{i}}}(s_{t}^{i},a_{t}^{i})\right),\text{clip}\left(\ell_{t}^{i}\left(\phi_{i}\right),1-\in ,1+\in \right){\cdot A}_{\pi_{\phi_{i}}}(s_{t}^{i},a_{t}^{i})\right],$$ (10)

where


$$\ell_{t}^{i}\left(\phi_{i}\right)=\pi_{\phi_{i}}\frac{\left(a_{t}^{i}|o_{t}^{i}\right)}{\pi_{\phi_{i}}^{\text{old}}(a_{t}^{i}|o_{t}^{i})}.$$ (11)

Here, $\pi_{\phi_{i}}(a_{t}^{i}|o_{t}^{i})$ represents the probability of the *i*-th agent selecting action $a_{t}^{i}$ given its observation $o_{t}^{i}$ at timestep t. $\ell_{t}^{i}\left(\phi_{i}\right)$ denotes the probability ratio between the new and old policies for the actor. $A_{\pi_{\phi_{i}}}(s_{t}^{i}\text{,}a_{t}^{i})$ is the advantage function, computed via the GAE according to equation (6).

The objective function of the entire actor network, ${ℒ}_{\text{Actor}}(\pi_{\varphi })$, consists of the objective functions of individual actors and the entropy function of their policies, $S\left(\pi_{\phi_{i}}(a_{t}^{i}|o_{t}^{i})\right)$, incorporating batch normalization:


$${ℒ}_{Actor}\left(\pi_{\varphi }\right)=\frac{1}{Tn}\sum {\mathrm{\backslash nolimits}}_{t=1}^{T}\sum {\mathrm{\backslash nolimits}}_{i=1}^{n}{ℒ}_{A}^{i}\left(\pi_{\phi_{i}}\right)+\sigma \frac{1}{Tn}\sum {\mathrm{\backslash nolimits}}_{t=1}^{T}\sum {\mathrm{\backslash nolimits}}_{i=1}^{n}S\left(\pi_{\phi_{i}}\left(a_{t}^{i}|o_{t}^{i}\right)\right),$$ (12)

where σ is a hyperparameter that scales the entropy term, n represents the number of agents, and T denotes the maximum number of timesteps for agent exploration. The exploration phase consists of a fixed number of trajectories, each containing experience data from different timestep. The actor policy network optimizes the policy by maximizing ${ℒ}_{\text{Actor}}(\pi_{\varphi })$.

MAPPO adopts a centralized critic network, where the joint state $z_{t}$ of all agents is used as input for the critic network to estimate state values. Additionally, a clipping mechanism is introduced to limit the magnitude of value updates. The objective function of the critic network, ${\text{L}}_{Critic}(\theta )$, is defined as follows:


$${ℒ}_{\text{C}\text{ritic}}(\theta )=\frac{1}{T}\sum {\mathrm{\backslash nolimits}}_{t=1}^{T}\text{max}\left[{{(V}_{\theta }(z_{t})-\;{\;R}_{t})}^{2},{{(\text{clip}(V_{\theta }(z_{t})\text{,}V}_{\theta^{\prime }}(z_{t})-\in ,V_{\theta^{\prime }}(z_{t})+\in )-R_{t})}^{2}\right].$$ (13)

where $R_{t}$ represents the discounted reward, $V_{\theta }\left(z_{t}\right)$ and $V_{\theta^{\prime }}(z_{t})$ denote the value estimates of the joint state $z_{t}$ under the new and old policies, respectively. During critic network training, ${\text{L}}_{Critic}(\theta )$ is minimized to improve value estimation accuracy. In MAPPO, the actor network and the critic network are typically optimized separately, which helps mitigate interference between networks and stabilize training.

### 4.3 Recurrent multi-agent proximal policy optimization

In MAPPO, if agents have full observability or their decision-making is minimally dependent on historical observations, both the actor and critic networks can be implemented using a multi-layer perceptron (MLP). However, in the multi-robot scheduling environment of the riveting and welding work cell studied here, agents operate under partially observable conditions. Additionally, after completing a riveting and welding task, the gripping robot must return to its initial position to grip the next stiffener plate. Therefore, the welding robots need to analyze historical data to determine whether the gripping robot is in a ready-to-cooperate state. MAPPO, as an on-policy algorithm, can only utilize data generated by the current policy during policy optimization. Unlike off-policy algorithms that utilize an experience replay mechanism, MAPPO cannot efficiently reuse past experiences. To address multi-agent scheduling in partially observable environments, a recurrent neural network (RNN) is integrated into the MAPPO framework. The actor and critic networks are built using RNNs, which increase the utility of individual samples by capturing more information from historical hidden states. This allows agents to effectively utilize historical information when handling temporal sequences and partially observable environments, enhancing policy optimization.

rMAPPO needs to retain the hidden states of the RNN when collecting data, as the RNN’s output depends on its past hidden states. In rMAPPO, the actor network employs an RNN to process the current observation $o_{t}^{i}$ along with the previous hidden state $h_{t-1}^{i}$, which is then used to generate the action probability distribution:


$$p_{t}^{i}\text{,}h_{t}^{i}=\pi_{\phi_{i}}\left(a_{t}^{i}|o_{t}^{i}\text{,}h_{t-\text{1}}^{i}\right),$$ (14)

where $p_{t}^{i}$ represents the action probability distribution produced by the corresponding actor network, and $h_{t}^{i}$ is the updated hidden state of the RNN layer, which retains historical observation information.

The critic network also incorporates an RNN to integrate historical information. It computes the state value estimation $V_{\theta }(z_{t})$ based on the global observation state $z_{t}$ and the previous hidden state $h_{t-\text{1,}V}$, while simultaneously updating the hidden state of the RNN layer $h_{t,V}$:


$$V_{\theta }\left(z_{t}\right),\;h_{t,V}=\pi_{\theta }\left(z_{t},h_{t-1,V}\right).$$ (15)

In the local observation processing unit of the actor network, the RNN processes past hidden states, enabling agents to incorporate past observations and address decision-making challenges in partially observable environments. In the state value estimation unit of the critic network, the RNN processes global states and past information, providing more accurate state value estimates to guide policy optimization.

In cooperative partially observable Markov decision processes (POMDPs), the convergence of rMAPPO is supported by two key theoretical foundations: the convergence of the centralized critic network and the synchronized policy updates across agents. According to the POMDP policy gradient theorem, the critic must first converge to the true team value function $V_{\theta }(S)$ to ensure the unbiased estimation of advantages. This requirement is satisfied through centralized training under the Robbins-Monro conditions [37]. When all agents synchronously update their policies using the clipped objective in PPO, the joint policy improvement is monotonic. This is because the shared critic enables consistent credit assignment among agents, while recurrent policy gradient updates preserve the Markov property of belief states when sufficient historical information is available [33,38,39]. Although the joint policy space is formally the Cartesian product of individual policy spaces, the effective space is a strict subset due to policy coupling and the shared team objective. Given that the team return $J\left(\pi_{\varphi }\right)$ is continuous and bounded over this space, and under the assumption of compactness and appropriate learning rates, the joint policy $\pi_{\varphi }$ can converge to a locally Pareto-optimal solution. Thus, the convergence of rMAPPO arises from the interplay between a stable value estimator and coordinated policy improvement among agents.

During the process of using rMAPPO to solve the robot scheduling problem, certain environmental constraints impose predefined action constraints on agents in specific scenarios. In such cases, the environment enforces the action directly, bypassing the neural network’s action generation. To handle this, action masking is employed to mask out actions generated by the actor network. For instance, when a welding robot and a gripping robot—in a collaborative state—reach a designated welding position, the environment imposes a waiting period for a certain number of timesteps to simulate the riveting and welding process. During these timesteps, action masking is applied, disabling actions generated by the actor network. Similarly, after a gripping robot completes a collaborative task, it must return to the initial position to grip a new stiffener plate. Since this movement sequence is explicitly defined in the environment, action masking is applied to prevent the actor network from generating actions during the corresponding timesteps. All such timesteps are treated as invalid timesteps during network computation. After masking invalid actions, it is essential to ensure that these invalid timesteps are excluded from loss calculation and gradient updates.

By introducing the mask $M_{t}^{i}$, invalid timesteps are excluded from the objective function computation. In rMAPPO, the per-agent objective function of the actor network ${ℒ}_{A}^{i}\left(\pi_{\phi_{i}}\right)$ is formulated as follows:


$${ℒ}_{A}^{i}\left(\pi_{\phi_{i}}\right)=\left[\;M_{t}^{i}\cdot \text{min}\left(\ell_{t}^{i}\left(\phi_{i}\right){\cdot A}_{\pi_{\phi_{i}}}\left(s_{t}^{i},a_{t}^{i}\right)\right)\text{,clip}\left(\ell_{t}^{i}\left(\phi_{i}\right),1-\in ,1+\in \right){\cdot A}_{\pi_{\phi_{i}}}(s_{t}^{i},a_{t}^{i})\right].$$ (16)

The overall objective function of the Actor network is still given by equation (12).

Accordingly, in the critic network, after applying a mask to the joint state $z_{t}$, the objective function ${\text{L}}_{\text{C}\text{ritic}}(\theta )$ is revised as follows:


$${ℒ}_{\text{C}\text{ritic}}(\theta )=\frac{1}{T}\sum {\mathrm{\backslash nolimits}}_{t=1}^{T}\text{max}\left[{{(V}_{\theta }\left(z_{t}^{\text{ma}\text{s}\text{k}}\right)-R_{t})}^{2},{{(\text{clip}(V_{\theta }(z_{t}^{\text{ma}\text{s}\text{k}})\text{,}V}_{\theta^{\prime }}(z_{t}^{\text{ma}\text{s}\text{k}})-\in ,V_{\theta^{\prime }}(z_{t}^{\text{ma}\text{s}\text{k}})+\in )-R_{t})}^{2}\right],$$ (17)

where $z_{t}^{\text{ma}\text{s}\text{k}}$ denotes the masked joint state, defined as: $z_{t}^{\text{ma}\text{s}\text{k}}=M_{t}^{i}⊙z_{t}$, where ⊙ represents the element-wise multiplication operation. During backpropagation, as the mask is already incorporated into the objective function, data from invalid timesteps is excluded from gradient computation, thus avoiding disruptions in the optimization process.

**Fig 9:**
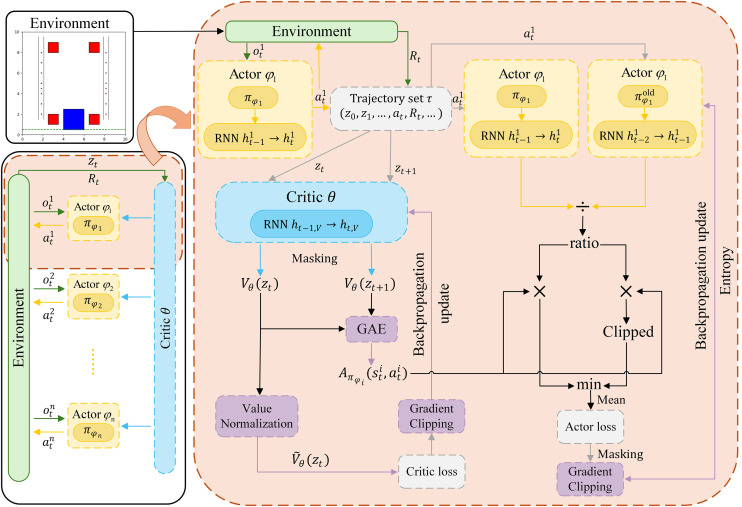
Framework of rMAPPO.

The rMAPPO algorithm framework is depicted in Fig 9, providing a detailed representation of the algorithm’s structure and execution process. The left module describes the interaction between agents and the environment, including the process of generating observations $o_{t}^{i}$, actions $a_{t}^{i}$, and discounted rewards $R_{t}$. The right module describes the detailed process by which the rMAPPO algorithm utilizes the aforementioned information. The critic network takes the joint state $z_{t}$ as input, along with the previous hidden state $h_{t-1,V}$ from its RNN, and outputs the global value function estimation $V_{\theta }(z_{t})$ at the current timestep. The critic network then updates the hidden state $h_{t,V}$ through the RNN module, and $V_{\theta }(z_{t})$ is forwarded to the GAE module for advantage estimation. The critic’s error is optimized via backpropagation using a clipped loss function. The actor network receives the local observation $o_{t}^{i}$ and the previous hidden state $h_{t-1}^{i}$, then outputs the action probability distribution $\pi_{\phi_{i}}$( $a_{t}^{i}|o_{t}^{i},h_{t-1}^{i}$). A clipping mechanism is applied to regulate updates, ensuring policy stability. Additionally, parameter updates in both the critic and actor networks are applied only to valid states and actions. Action masking is used to filter out invalid information, mitigating its impact on training stability.

In multi-agent scenarios, the joint value function may exhibit significant variations due to the complexity of states and actions. Value normalization mitigates this issue by constraining the value function within a narrower range. This improves gradient stability during training, enabling the critic network to align more effectively with its optimization objective and thus accelerating convergence. The normalized value function ${\tilde{V}}_{\theta }(z_{t})$ is defined as:


$${\tilde{V}}_{\theta }\left(z_{t}\right)=\frac{\left(V_{\theta }\left(z_{t}\right)-\hat{\mu }\right)}{\sqrt{\hat{\nu }-{\hat{\mu }}^{2}}},$$ (18)

where $\hat{\mu }$ and $\hat{\nu }$ represent the updated mean and squared mean, respectively [40].

### 4.4 Reward function

The reward function provides a quantitative assessment of the utility of an agent’s action in a given state. A well-designed reward function can enhance the critic’s estimation accuracy. To enable efficient cooperation among agents, and to ensure the successful execution of stiffener plate riveting and welding tasks, the reward functions are defined separately. They are tailored for two distinct agent types: gripping robots and welding robots, as detailed below.

1. Gripping robot: The reward function $r_{0}$ for the gripping robot consists of two terms. The first is the negative sum of absolute differences between its y-coordinate and those of the remaining riveting positions. The second is a constant reward term η. To encourage cooperation among robots, a fixed reward η is assigned during the non-cooperative period, when the gripping robot returns to the origin to grip the next stiffener plate after completing the previous riveting task. When the gripping robot is in a ready-to-cooperate state, η is set to 0. The reward function $r_{0}$ is formulated as follows:

$$r_{0}=-\sum {\mathrm{\backslash nolimits}}_{i=0}^{G_{\text{res}}}k_{0}\cdot \left|d_{{\text{A}}_{0}}-d_{{\text{target}}_{i}}\right|+\eta ,$$ (19)

where $k_{0}$ is a constant, $G_{\text{res}}$ represents the number of stiffener plates yet to be riveted, $d_{{\text{A}}_{0}}$ is the y-coordinate of the gripping robot, $d_{{\text{target}}_{i}}$ is the y-coordinate of the *i*-th stiffener plate.

1. Welding robot: the reward functions $r_{1}$, $r_{2}$, $r_{3}$, $r_{4}$ for the four welding robots are determined by the gripping robot’s y-coordinate and a cooperation reward. The reward function is defined as follows:

$${r_{i}=-\;k}_{0}\cdot \left|d_{{\text{A}}_{0}}-d_{{\text{A}}_{i}}\right|+\left(\text{1}-\frac{G_{\text{res}}}{G_{\text{total}}}\right)\cdot \xi \text{,}$$ (20)

where $d_{{\text{A}}_{i}}$ is the y-coordinate of the *i*-th welding robot, $G_{\text{total}}$ is the total number of stiffener plate welding tasks. When the welding robot is in a cooperative state, ξ is a positive value; otherwise, ξ = 0. The cooperation reward ξ for welding robots is designed to improve the overall efficiency of completing the welding tasks.

Since the research scenario involves multi-agent collaboration, a shared reward mechanism is employed. The final reward at timestep t, denoted as $r_{t}$, is the cumulative reward of all agents within the same timestep, with additional penalties for elapsed timesteps and unfinished tasks. The reward function $r_{t}$ is formulated as follows:


$$r_{t}=\sum {\mathrm{\backslash nolimits}}_{i=0}^{4}r_{i}-\frac{t}{T}\cdot k_{1}-G_{\text{res}}\cdot k_{2},$$ (21)

where $k_{\text{1}}$ and $k_{\text{2}}$ are constants. A well-designed penalty can significantly enhance coordination efficiency among agents. However, if the penalty is too large, it may diminish agents’ motivation to complete tasks efficiently.

## 5 Result analysis and digital twin implementation

This section first describes the experimental environment setup, including the employed equipment and the chosen parameters. An ablation study is then conducted on the key components of the rMAPPO framework to evaluate the effectiveness of the proposed improvements. Subsequently, the performance of rMAPPO is compared and analyzed against other multi-agent reinforcement learning baseline algorithms. Finally, the simulation results of the rMAPPO model are integrated with and evaluated using the digital twin platform to assess its implementation and effectiveness.

### 5.1 Experimental setting

During the training phase, the servo-controlled base of the gripping robot is set to a movement speed of 2 m/s, while the servo-controlled bases of the welding robots are set to 3 m/s. The environment timestep is defined as Δ*t* = 0.1s, with a collaboration duration of 20 Δ*t*, and the gripping robot’s stiffener plate gripping time at its initial position set to 10 Δ*t*. The maximum length of H-beams that can be processed at the work cell is 8 meters. Based on actual production conditions, each H-beam typically requires 6–10 stiffener plates, with a minimum spacing of 0.2 meters between adjacent plates. Accordingly, five groups of experiments are designed, with the number of stiffener plates set to 6, 7, 8, 9, and 10, respectively. Each experimental group is repeated 5 times, and the evaluation metrics are averaged. In each episode, the stiffener plate positions are randomly generated within a range of 0–8 meters.

All experiments are conducted on a workstation featuring an Intel Core i9-13980HX CPU and an NVIDIA RTX 4060 GPU. The software versions used are as follows: python(3.8), pytorch (1.5.1), Unity 3D (2021.3.36f1c1), CUDA (10.1). Details regarding the learning rates for the actor and critic networks, along with the clipping parameter ε, will be provided in subsequent sections. Unless otherwise stated, parameter settings are shown in Table 1. The training procedure consists of 80 episodes, each with a maximum length of 2500 timesteps.

**Table 1:** Simulation settings of rMAPPO algorithm.

Parameters	Value
Reward function parameter $k_{0}$	0.6
Reward function parameter η	1
Reward function parameter ξ	1
Reward function parameter $k_{1}$	1
Reward function parameter $k_{2}$	0.05
Discount factor γ	0.99
GAE λ	0.95
Mini-batch size	2
Hidden layer size	128
Gain	0.01
Entropy coefficient	0.01
Recurrent layers number	1

### 5.2 Convergence performance

Root mean square propagation(RMSprop) is adopted to adaptively adjust the learning rates of the actor and critic networks during training. RMSprop adjusts learning rates by applying an exponentially weighted moving average, decreasing the rate for parameters with large gradients and increasing it for those with smaller ones. This optimizer is well-suited for algorithms involving temporal models and non-stationary optimization tasks.

A proper initial learning rate for RMSprop can significantly improve convergence speed and stability. One core feature of PPO-based algorithms is the clipping parameter ε, which controls policy updates and is typically set between 0.1 and 0.3 based on empirical evidence [33]. To determine appropriate initial learning rates for the actor and critic networks, as well as the clipping parameter ε, five experimental groups are configured. Each experimental group is repeated 5 times with different random seeds. The mean cumulative reward of the final 10 episodes is used as the performance metric. If training does not converge, the average reward is recorded as 0.

**Fig 10:**
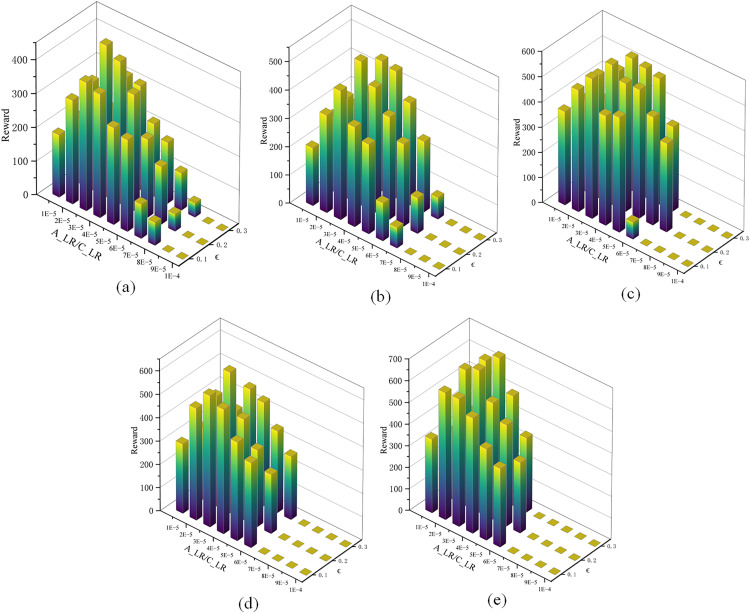
Initial learning rate and clipping parameter selection. In (a), (b), (c), (d) and (e), different numbers of stiffener plates are designated for riveting and welding on the H-beam, with positions randomly assigned.

As shown in Fig 10, all subfigures (a-e) demonstrate that the reward values are sensitive to the parameter combinations. The color gradient from blue to yellow indicates the presence of distinct performance peaks. Across the five experiments, as the number of stiffener plates increases, the task becomes more complex. Accordingly, the distribution of high-reward regions (yellow bars) shifts. Based on the results of all five experiments, lower initial learning rates ensure more stable updates and lead to higher cumulative rewards. Therefore, the initial learning rates for the actor and critic are set to (A_LR/C_LR=3e−5/3e−5), and the clipping parameter ∈ is set to 0.2 for the remaining performance evaluations, as this configuration consistently achieves convergence and yields the highest rewards.

### 5.3 Ablation experiment and algorithm comparison

The ablation experiment is conducted for rMAPPO by removing the RNN layers from the actor and critic networks. This evaluates the role of the recurrent module in rMAPPO under a partially observable multi-agent scenario. In the MAPPO network, which replaces the RNN layers with a multi-layer perceptron (MLP), the relevant parameters of the actor and critic are kept consistent with those in rMAPPO.

**Fig 11:**
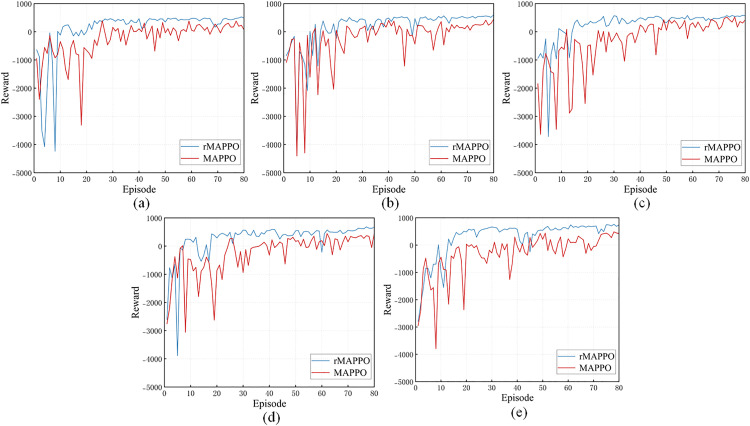
Convergence performance of rMAPPO and MAPPO. In (a), (b), (c), (d) and (e), different numbers of stiffener plates are designated for riveting and welding on the H-beam, with positions randomly assigned.

Fig 11 illustrates the convergence performance curves of rMAPPO and MAPPO. Experimental results indicate that increasing the number of stiffener plates leads to greater task complexity, resulting in more pronounced training instability in MAPPO. In contrast, rMAPPO demonstrates stronger adaptability, outperforming MAPPO in both convergence speed and reward accumulation. It reaches a stable state 10–15 episodes earlier and achieves higher steady-state reward values. The experimental results indicate that rMAPPO better captures the temporal dependencies of the environment. Particularly in the later training stages, the RNN module enhances agents’ ability to retain historical information, enabling them to learn superior policies. This highlights rMAPPO’s stronger adaptability to sequential dependencies in partially observable environments.

**Fig 12:**
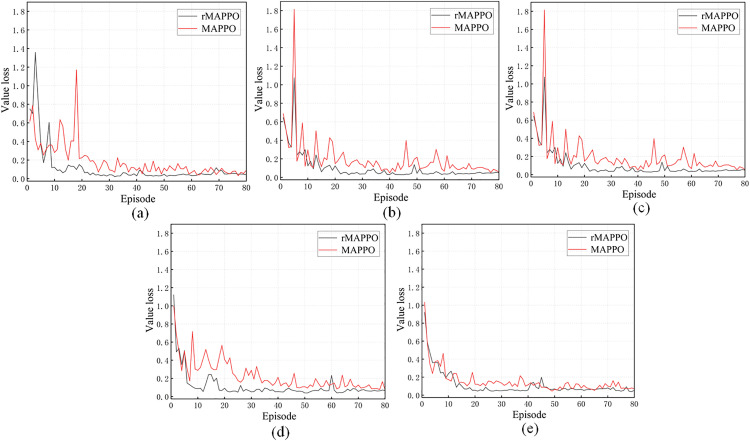
Value loss of rMAPPO and MAPPO. In (a), (b), (c), (d) and (e), different numbers of stiffener plates are designated for riveting and welding on the H-beam, with positions randomly assigned.

Fig 12 depicts the value loss trends of rMAPPO and MAPPO across the five experimental groups. Clear differences in training dynamics are observed between rMAPPO and MAPPO. Across all five subfigures, both algorithms exhibit value loss curves that transition from high volatility to gradual convergence. However, rMAPPO shows more stable convergence in most cases. In the early training stages, both algorithms experience significant oscillations in value loss. As training progresses (around episodes 20–50), rMAPPO’s loss curve declines more rapidly, with reduced fluctuations, and eventually stabilizes around 0.1–0.2 after episode 50. In subfigure (e), where the number of stiffener plates is highest, rMAPPO demonstrates superior stability. This indicates a more robust value function estimation capability when facing increased task complexity. Such performance gain can be attributed to the RNN module in rMAPPO, which captures temporal dependencies in the critic network and significantly enhances estimation accuracy.

**Fig 13:**
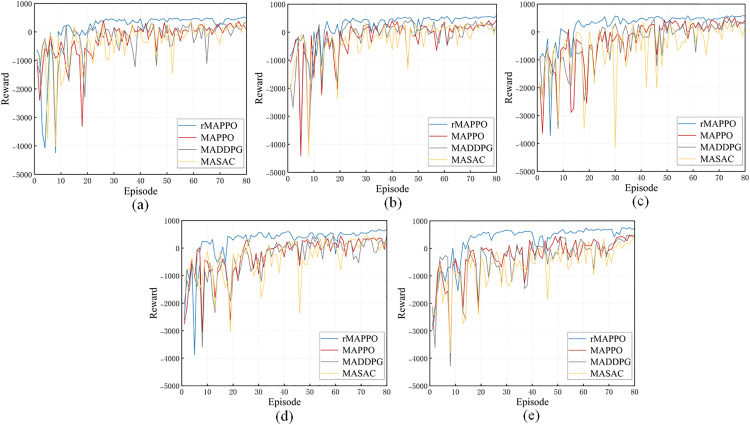
Comparison of convergence performance of rMAPPO with other baseline methods. In (a), (b), (c), (d) and (e), different numbers of stiffener plates are designated for riveting and welding on the H-beam, with positions randomly assigned.

The convergence performance and cumulative rewards of the proposed rMAPPO algorithm are further evaluated in comparison with two baseline methods: MADDPG and MASAC. Due to differences in task characteristics and the design of the reward function, the maximum achievable reward varies across different riveting and welding scenarios. As illustrated in Fig 13, five comparative experiments were conducted. With increasing task complexity—represented by a growing number of stiffener plates from subfigures (a) to (e)—the performance differences among the algorithms become more pronounced. In all cases, rMAPPO consistently achieves the highest cumulative rewards, demonstrating stronger generalization and reaching optimal performance earlier than other algorithms. Its final convergence performance demonstrates a clear advantage over the baselines, especially as task complexity increases. Moreover, rMAPPO exhibits greater training stability. It enters the stable improvement phase more quickly, maintains smoother training, and adapts more effectively to fluctuations caused by exploration.

**Fig 14:**
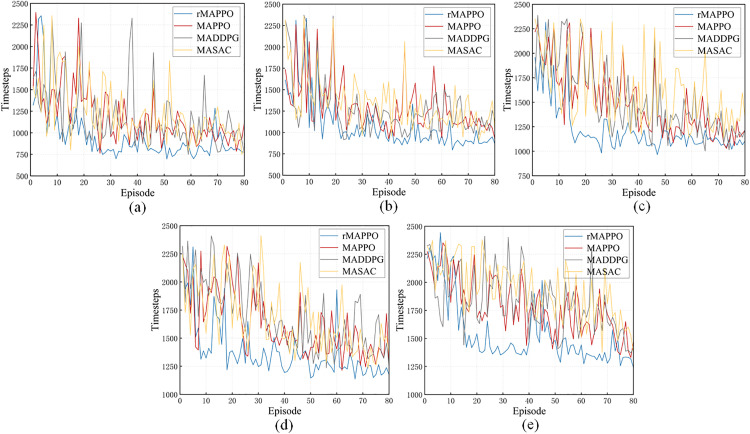
Comparison of timesteps of rMAPPO with other baseline methods. In (a), (b), (c), (d) and (e), different numbers of stiffener plates are designated for riveting and welding on the H-beam, with positions randomly assigned.

Across the five experimental groups, the completion time for different riveting and welding tasks varies depending on task complexity. As shown in Fig 14, the rMAPPO algorithm demonstrates a clear advantage in time efficiency. Throughout the five experiments with increasing task complexity, it consistently requires the fewest timesteps. From subfigure (a) to (e), all algorithms show a general increase in timestep consumption. However, rMAPPO shows the slowest growth in timestep consumption and the smallest fluctuation range, indicating excellent stability. Upon entering the convergence phase, it completes operations in just 700–900 timesteps for most simpler tasks (subfigures a and b). For more complex tasks, it still performs significantly better than other algorithms, requiring only 1200–1400 timesteps. Moreover, rMAPPO converges faster during training, reaching lower timestep levels earlier. This reflects its high efficiency and robustness in handling randomly distributed riveting and welding tasks. Overall, these results highlight rMAPPO’s clear advantages in both stability and policy quality, particularly in complex or partially observable environments.

### 5.4 Digital twin implementation

In the testing phase, the trained model is deployed on the industrial computer and integrated with both the physical work cell and its digital twin platform for joint debugging. This aims to assess the impact of the rMAPPO model on production efficiency when integrated with the digital twin system. All experiments involving the digital twin are conducted in Unity 3D.

This study examines the system’s real-time performance in practical deployment. During task execution, real-time latency primarily stems from three sources: communication, computation, and physical delays [41]. Testing shows that the rMAPPO model incurs a decision-making delay of approximately 10 ms when generating scheduling strategies. The physical delay of the servo drives or mechanical joints is around 3 ms. Since the rMAPPO model is deployed on the industrial computer, the scheduling policy is translated into servo base control commands and dispatched to both the digital twin platform and the physical work cell. In the physical work cell, the industrial computer sends six-axis robot control or servo base control commands to drive the robots. Meanwhile, the digital twin platform receives the six-axis motion data from the physical robots via the industrial computer. Combined with the transmitted servo base control actions, this data enables the twin robot to execute corresponding actions in synchronization. The digital twin platform can also transmit monitoring signals back to the industrial computer. The average end-to-end transmission delays between modules, as measured in tests, are shown in Table 2. After the industrial computer processes and dispatches the generated scheduling policy to both the physical and virtual systems, transmission delays occur as the six-axis data is returned to the digital twin platform for execution. However, physical delays arise during the actual execution of robotic actions. These combined effects reduce the execution time gap between virtual and physical robots, improving synchronization. Ultimately, system latency remains within a few milliseconds, meeting the real-time requirements of riveting and welding tasks.

**Table 2:** End-to-end data transmission delay.

Category	Data	Delay(ms)
Industrial PC to digital twin platform	Servo base control actions	2.027
Digital twin platform to industrial PC	Monitoring signals	1.963
Industrial PC to work cell	Robot control commands	2.211
Work cell to digital twin platform	Six-axis data	4.293

Under the integration of the digital twin and physical work cell, ten production rounds were conducted using different methods for five task groups, each with varying stiffener quantities and positions. The performance of rMAPPO is assessed and compared with other baseline algorithms, as well as with the fixed policy previously implemented in the work cell. The fixed policy operates based on a predefined sequence: the gripping robot first moves to the nearest riveting and welding target, then signals the nearest welding robot to arrive and jointly executes the riveting and welding task. Afterward, the gripping robot returns to the initial position to retrieve the next stiffener plate and repeats the process.

**Fig 15:**
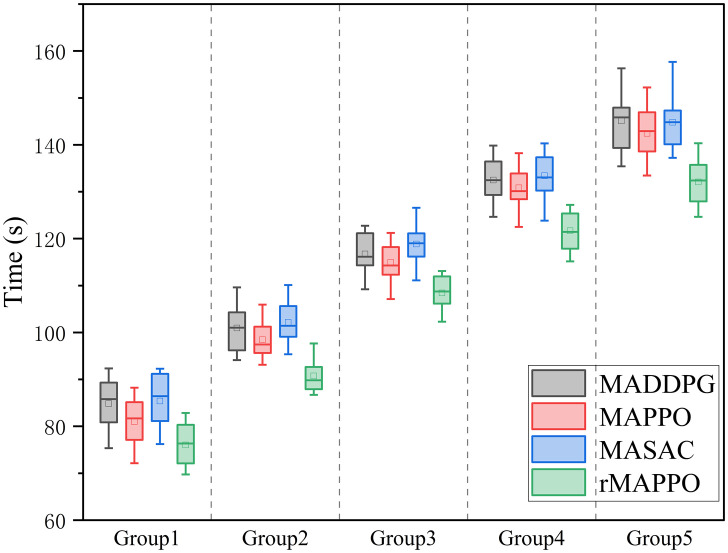
Completion times for different algorithms.

**Fig 16:**
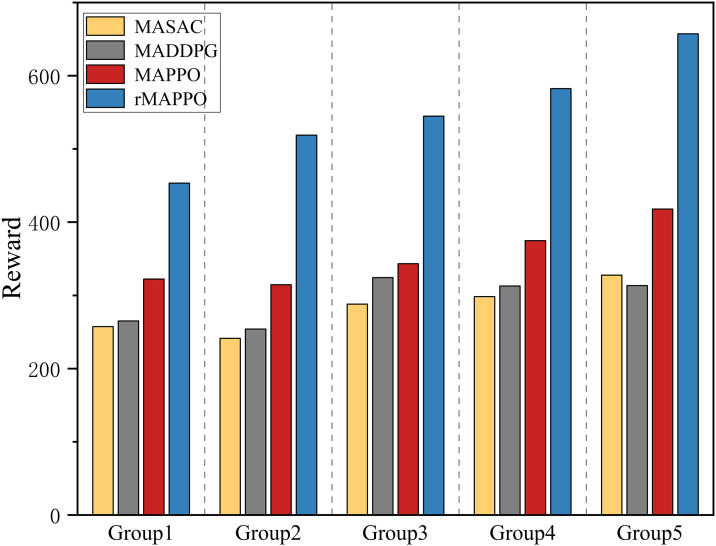
The average cumulative reward of algorithms.

Fig 15 compares the task completion times of four multi-agent reinforcement learning algorithms across five task groups of increasing complexity using box plots. Overall, as task complexity increases, all algorithms show a rising trend in execution time. Among them, rMAPPO consistently achieves the shortest completion times and greater stability in most groups, maintaining relatively low median times (approximately 75–110 seconds). In the integrated testing environment involving both the physical riveting and welding work cell and the digital twin platform, rMAPPO reduces task completion time by approximately 7% to 10% compared to other reinforcement learning algorithms. Fig 16 illustrates the average cumulative rewards for each algorithm across the five testing groups. rMAPPO outperforms all others in every group, indicating stronger overall performance and better compatibility with the reward function design adopted in this study.

Table 3 compares the average task completion times between reinforcement learning algorithms and a rule-based fixed policy across five experimental groups. In the integrated testing environment involving the physical riveting and welding work cell and its digital twin platform, all reinforcement learning methods exhibit improved efficiency over the fixed policy. Among them, rMAPPO achieves the most significant reduction in task duration, decreasing actual execution time by 12.4% to 15.8% compared to the fixed policy. Based on both the training performance in the 2D multi-agent interaction environment and the evaluation results in the physical–digital integrated system, rMAPPO exhibits greater stability and efficiency. As such, it is more suitable for solving the robotic scheduling problem in the H-beam riveting and welding work cell.

**Table 3:** The average completion time of each method.

Algorithm	Group 1		Group 2		Group 3		Group 4		Group 5	
	Time(s)	%	Time(s)	%	Time(s)	%	Time(s)	%	Time(s)	%
Fixed policy	90.31	–	106.93	–	124.55	–	138.93	–	152.42	–
rMAPPO	76.02	15.8	90.74	15.1	108.44	12.9	121.69	12.4	132.13	13.3
MAPPO	81.01	10.3	98.48	7.9	114.93	7.7	130.85	5.8	142.51	6.5
MADDPG	84.83	6.1	100.96	5.6	116.73	6.3	132.49	4.6	145.14	4.8
MASAC	85.41	5.4	102.17	4.5	118.87	4.6	133.44	3.9	144.83	5.0

## 6 Conclusion

This paper proposes an intelligent scheduling method for robots in the H-beam riveting and welding work cell. RNNs are incorporated into the actor and critic networks, enabling agents to effectively retain historical states and engage in long-term policy learning under partial observability. Furthermore, a digital twin simulation platform is developed for the riveting and welding work cell, offering a safe and efficient environment for validating reinforcement learning decisions. Under a well-designed reward function and an effective parameter update mechanism, the rMAPPO model’s scheduling decisions have been validated through experiments on both the physical and digital twin work cells, leading to reduced robot idle time and improved production efficiency. In the future, we will extend the application of the proposed method to a wider range of industrial scenarios.

## Supporting information

S1 File Data_and_model_files. (ZIP)
